# Soft tissue substitutes in non-root coverage procedures: a systematic review and meta-analysis

**DOI:** 10.1007/s00784-016-2044-4

**Published:** 2017-01-20

**Authors:** Kristina Bertl, Maximilian Melchard, Nikolaos Pandis, Michael Müller-Kern, Andreas Stavropoulos

**Affiliations:** 1 Department of Periodontology, Faculty of Odontology, University of Malmö, Carl Gustafs väg 34, 20506 Malmö, Sweden; 2 Division of Oral Surgery, School of Dentistry, Medical University of Vienna, Vienna, Austria; 3 Department of Orthodontics and Dentofacial Orthopedics, Dental School/Medical Faculty, University of Bern, Bern, Switzerland; 4 Division of Conservative Dentistry and Periodontology, School of Dentistry, Medical University of Vienna, Vienna, Austria

**Keywords:** Attached gingiva, Keratinized tissue, Meta-analysis, Randomized controlled trials, Soft tissue augmentation, Systematic review

## Abstract

**Objectives:**

The present systematic review compared the effectiveness of soft tissue substitutes (STSs) and autogenous free gingival grafts (FGGs) in non-root-coverage procedures to increase keratinized tissue (KT) width around teeth.

**Materials and methods:**

Included studies fulfilled the following main eligibility criteria: (a) preclinical in vivo or human controlled trials using FGG as control, (b) non-root-coverage procedures, and (c) assessment of KT width. Meta-analysis was performed on the gain in KT width (primary outcome variable) and several secondary variables.

**Results:**

Eight human trials with short observation time evaluating five different STSs were identified. FGG yielded consistently significantly (*p* < 0.001) larger increase in KT width irrespective whether the comparison regarded an acellular matrix or a tissue-engineered STS. Further, FGG yielded consistently ≥2 mm KT width postoperatively, while use of STS did not, in the few studies reporting on this outcome. On the other hand, STSs resulted in significantly better aesthetic outcomes and received greater patient preference (*p* < 0.001).

**Conclusions:**

Based on relatively limited evidence, in non-root-coverage procedures, FGG (1) resulted consistently in significantly larger increase in KT width compared to STS and (2) yielded consistently ≥2 mm KT width postoperatively, while STSs did not. STSs yielded significantly better aesthetic outcomes, received greater patient preference, and appeared safe.

**Clinical relevance:**

Larger and more predictable increase in KT width is achieved with FGG, but STSs may be considered when aesthetics is important. Clinical studies reporting relevant posttreatment outcomes, e.g., postop KT width ≥2 mm, on the long-term (>6 months) are warranted.

**Electronic supplementary material:**

The online version of this article (doi:10.1007/s00784-016-2044-4) contains supplementary material, which is available to authorized users.

## Background

It is currently accepted that a minimum width of keratinized tissue (KT) around teeth is not necessary to maintain periodontal health and/or prevent gingival recession development, when adequate plaque control is performed. However, if plaque control is inadequate and/or a submarginal restoration is necessary, a minimum of 2 mm of KT (i.e., ca. 1 mm of free gingiva and 1 mm attached gingiva (AG)) is recommended [1]; hence, in such patients lacking 2 mm KT width, soft tissue augmentation procedures should be considered (for review, see Scheyer et al. [2]).

Various non-root coverage procedures aiming to increase the width of KT in terms of apico-coronal dimension have been proposed through the years. These include various flap designs, usually in combination with autogenous soft tissue grafting. In a review performed a few years ago by Thoma et al. [3] the apically positioned flap (APF) in combination with an autogenous free gingival graft (FGG) from the palate was found to result in significantly higher increase in KT width compared to APF alone and marginally significant higher increase compared to APF in combination with a soft tissue substitute (STS). Grafting with FGG, however, has some major disadvantages: (1) need for second surgical site contributing to patient morbidity, (2) occasionally relatively limited supply, (3) some risk for surgical complications (i.e., intraoperative violation of the greater palatine vessels and nerves or a strong postoperative bleeding), and (4) often an unsatisfactory aesthetic outcome due to a “patch-like” appearance with significant color mismatch to the neighboring tissue. Thus, STSs appear as an attractive alternative to FGG.

Indeed, new STS products have appeared in the market since the review mentioned previously [3], and although all alternatives to a FGG have been summarized in the last AAP workshop [1], no recent meta-analysis is available on this specific comparison. Hence, the aim of the present study was to conduct a systematic review and meta-analysis to answer the following focused question, according to the population, intervention, comparison, outcomes, and study design criteria [4]: “In animal or human trials, are STSs equally efficacious as autogenous palatal soft tissue grafts (FGG or connective tissue grafts (CTG)) in non-root-coverage procedures aiming to increase the apico-coronal width of KT around teeth, including aesthetic and patient-reported outcome measures (PROMs)?”

## Materials and methods

### Protocol and eligibility criteria

The present systematic review followed the Preferred Reporting Items for Systematic Reviews and Meta-analyses (PRISMA; Appendix 1; [5, 6]). The following inclusion criteria were applied during literature search on original studies: (a) English or German language, (b) full text available, (c) preclinical in vivo trials or (d) human controlled or randomized controlled clinical trials (RCTs) with ≥5 patients and ≥3-month follow-up, (e) non-root coverage procedures, and (f) preoperative and postoperative assessment of the KT width. Studies were excluded if not all inclusion criteria were met and if they regarded in vitro studies or augmentation of KT in fully edentulous patients or around implants.

### Information sources and literature search

Electronic search was performed on three sources (last search December 31, 2015; no date restriction used): Medline (PubMed), Embase (Ovid), and CENTRAL (Ovid). The database Medline (PubMed) was searched with the following keywords: (acellular dermal matrix OR dermal matrix allograft OR alloderm OR soft tissue graft OR free gingival graft OR human fibroblast-derived dermal substitute OR dermagraft OR apligraf OR collagen matrix OR extracellular membrane OR gingival autograft OR soft tissue augmentation OR soft tissue transplantation OR soft tissue correction) AND (keratinized tissue OR keratinized gingiva OR attached gingiva OR attached mucosa OR keratinized mucosa). For the other two databases, comparable terms were used but modified to be suitable for specific criteria of the particular database. Additionally, grey literature (conference abstracts and www.opengrey.eu) was browsed and a “manual search” through the electronically available material of the following relevant journals, including publications ahead of print, was performed: *Journal of Clinical Periodontology*, *Journal of Periodontology*, *Journal of Periodontal Research*, *Clinical Oral Investigations*, *Journal of Dental Research*, and *Parodontologie.* Screening of the reference lists of previous reviews and selected full texts was also conducted. Finally, a forward search via Science Citation Index of included papers was added and ClinicalTrials.gov was checked on unpublished or ongoing studies.

### Data collection and extraction

Two authors (KB, MM) independently checked titles, abstracts, and finally full texts with regard to the predefined eligibility criteria. Abstracts with unclear methodology were included in full-text assessment to avoid exclusion of potentially relevant articles. One author (KB) repeated the literature search. In case of ambiguity, consensus through discussion was achieved together with a third author (AS).

Two authors (KB, MM) extracted twice the following data (if reported): width of KT at baseline and after 3, 6, and 12 months and/or KT gain, difference in KT gain, and graft contraction; frequency of postintervention width of KT ≥2 mm; and aesthetic (i.e., tissue color and texture) and PROMs (i.e., postoperative pain level and patient preference/satisfaction).

### Risk of bias assessment

Two authors (MM, MMK) independently evaluated the risk of bias applying the Cochrane Collaboration’s Tool for assessing risk of bias (Cochrane Handbook for Systematic Reviews of Interventions) [7]. The following domains were evaluated at “low,” “high,” or “unclear” risk of bias: (a) random sequence generation, (b) allocation concealment, (c) blinding of outcome assessment, (d) incomplete outcome data, (e) selective reporting, and (f) other bias. As the specific research question (comparison of an autologous palatal tissue graft with a STS) makes it impossible to blind the personnel during surgery and almost impossible to blind the patients, the criterion “blinding of participants and personnel,” originally included in the tool, was excluded herein. The overall risk of bias for an individual study was judged as follows: low, if all criteria were evaluated to be of low risk; high if at least one criterion was evaluated to be of high risk; and unclear, if at least one criterion was evaluated to be of unclear risk but no criterion of high risk. One author (MM) repeated the assessment, and in case of ambiguity, consensus through discussion was achieved.

### Synthesis of results

The postintervention mean difference between STS and autogenous palatal soft tissue graft groups, regarding gain in KT width (primary outcome variable) and several secondary variables [graft contraction, aesthetic outcome (i.e., tissue color and texture match to the neighboring tissue), and PROMs (i.e., pain level and preference/satisfaction)], was assessed by meta-analysis.

Clinical heterogeneity of included studies was gauged by assessing the treatment protocol, particularly participants and setting, materials used, timing of data collection, and measurement techniques. Statistical heterogeneity was assessed by graphic display and consistency of the estimated treatment effects from the included trials in conjunction with 95% confidence intervals (CIs). The chi-squared test was used to assess heterogeneity; a *p* value <0.1 would be considered indicative of significant heterogeneity [8]. *I* ^2^ test for homogeneity was also undertaken, where possible, to quantify the extent of heterogeneity prior to each meta-analysis.

Quantitative synthesis was performed using the DerSimonian and Laird random effect methods [9] for all included studies and separately for comparing “acellular graft substitutes vs. FGG” and “tissue-engineered graft substitutes vs. FGG.” A weighted mean pooled treatment effect was calculated with 95% CIs for the continuous outcome variables using a random effects model; a random effects model was considered more appropriate in view of the variation in population and settings. Pooled estimates were also calculated separately per follow-up period (i.e., 3, 6, and 12 months). Most comparisons (9/11) were derived from split-mouth studies, and in those instances where the standard deviation of the mean difference was not available, it was calculated using the formula

$$ \sqrt{sd\_{\mathrm{treat}}^2+sd\_{\mathrm{control}}^2-2\ast r\ast sd\_\mathrm{treat}\ast sd\_\mathrm{control}} $$

where sd_treat and sd_control are the corresponding standard deviations and *r* is the correlation coefficient for the between treatment group measurements. The correlation coefficient was set at 0.5; however, syntheses were also conducted using values of *r* = 0 in the context of sensitivity analyses. For binary outcomes (i.e., aesthetic outcome and PROMs), a similar adjustment was implemented using *r* = 0.5 to calculate the variances on a logarithmic scale, before conversion to the exponentiated form.

## Results

### Study selection

The flowchart of the literature search is presented in Appendix 2. Out of 485 originally identified studies, 314 and 139 were excluded based on title and abstract, respectively. Seven additional records were retrieved from reference lists of previous reviews and selected full-text articles, and two were identified from the forward search. No unpublished or ongoing studies were identified. From the 41 articles selected for full-text review, 33 [References of excluded studies, 1-33] were excluded for various reasons (for details, see Appendix 3). Finally, eight clinical trials [10–17] were included; further on, the studies will be cited with Roman numbers as indicated in Table 1.

**Table 1:** Characteristics of the included studies on non-root coverage procedures to increase the width of keratinized tissue

Study (year)	Study no.	Study design	No. of patients (m/f, age)	Test group			Control group		Treatment indication	Follow up period (months)	Loss to follow-up/training purpose (*n*)
			Smoking status	Product (no. of sites)	Graft width^a^ Graft length^b^	No. of layers (no. of patients)	Type of graft (no. of sites)	Graft width^a^ Graft length^b^ (no. of patients)			
Acellular matrices											
Wei et al. (2000) [17]	I	(R)CT	12 (7/5, range 25–79) NS	AD (6)	8.4–9.67 mm NR	1	FGG (6)	5.67–8.00 mm NR	Insufficient zone of AG (≤1 mm)	6	0/0
Harris (2001) [10]	II	(R)CT	45 (18/27, range 14–67) NR	AD (15)	NR	1	Control 1: FGG (15) Control 2: CTG (15)	NR NR	Insufficient zone of KT	3	0/0
Nevins et al. (2010) [15]	III	(R)CT, SM	6 (1/5, mean 41) NR	DM (6)	NR	1 (5) 2 (1)	FGG (6)	NR	Insufficient zone of AG (≤2 mm)	3	0/0
Nevins et al. (2011) [16]	IV	(R)CT, SM	5 (NR, range 20–70) NR	MG (5)	NR	1	FGG (5)	NR	Insufficient zone of AG (≤2 mm)	12	0/0
McGuire & Scheyer (2014) [14]	V	RCT, SM	30 (6/24, range 28.1–70.6) NS and FS (since at least 6 months)	MG (30)	“As widely as possible” NR	1	FGG (30)	4 mm NR	Insufficient zone of KT (<2 mm)	6	0/0
Tissue-engineered STSs											
McGuire & Nunn (2005) [11]	VI	RCT, SM	25 (9/16, range 27–56.5) 16 NS, 9 FS	DG (25)	5 mm NR	1 (5) 3 (15) 4 (2)	FGG (25)	5 NR	Insufficient zone of AG	12	0/3
McGuire et al. (2008) [12]	VII	RCT, SM	25 (8/17, range 31.1–69.7) 14 NS, 11 FS	CT (25)	5 mm NR	3	FGG (25)	5 NR	Insufficient zone of AG (≤1 mm)	6	0/0
McGuire et al. (2011) [13]	VIII	RCT, SM	96 (39/46, range 18.0–70.8) NS and FS (since at least 3 months)	CT (96)	5–20 mm 10–30 mm	3	FGG (96)	4 mm (94) 5 mm (2) 8–30 mm	Insufficient zone of AG (≤1 mm)	6	0/11

*AD* Alloderm®, *AG* attached gingiva, *CT* CelTx™ (Apligraf®), *CTG* connective tissue graft, *DG* Dermagraft®, *DM* DynaMatrix®, *FGG* free gingival graft, *FS* former smokers, *KT* keratinized tissue, *LD* Lyodura®, *MG* Mucograft®, *NR* not reported, *NS* non-smokers, *RCT* randomized controlled clinical trial, (*R*)*CT* according to authors randomized, but randomization process not defined, *SM* split mouth

^a^Width = apico-coronal dimension

^b^Length = mesio-distal dimension

### Study characteristics

#### Study populations

Sample size ranged from 5 to 96 patients; two studies (VI, VIII) excluded from the analysis some patients, which had initially been treated for training purposes. All studies reported patient age range, but one (III) reported mean age. Sex distribution was reported in seven studies (I–III, V–VIII). Smoking status was not reported in three studies (II–IV); four studies (V–VIII) included non-smokers and former smokers and one (I) only non-smokers (Table 1).

#### Type of intervention

Indication for treatment in all included studies was an insufficient zone of KT [two studies (II, V)] or of AG [six studies (I, III, IV, VI–VIII)]. All studies were RCTs [6 with split-mouth design (III–VIII)] and comparisons regarded “STSs and APF” vs. “FGG and APF.” The follow-up period ranged from 3 to 12 months. All studies reported no patient loss to follow-up (Table 1).

#### Type of autogenous soft tissue grafts and STSs

All studies used FGG as the main control group, while one study (II) included a second control group with subepithelial CTG. The apico-coronal graft dimension in the control group was either predefined to 4–5 mm (V–VIII) or measured during grafting (I).

Five different STSs were tested: three acellular matrices [AlloDerm® (I, II); DynaMatrix® (III); Mucograft® (IV, V)] and two tissue-engineered STSs [CelTx™ (VII, VIII); Dermagraft® (VI)] (Table 2). The apico-coronal dimension of the STS was either predefined (5 mm) (VI, VII) or measured during grafting (I, VIII). Four studies (I, II, IV, V) used the STS in a single layer and two studies (VII, VIII) in three layers, and two studies (III, VI) tested various numbers of layers (Table 1). In seven studies (I–VII) no remarkable adverse events (AE) were reported. One study (VIII) included a detailed AE report: in two patients, the polycarbonate membrane on which CelTx™ is supplied was unintentionally used; a third patient showed a mouth ulceration; another three serious AE (e.g., pneumonia, chest pain) were considered unrelated to the intervention. Currently, Dermagraft® is not available in the market and CelTx™ is not distributed for dental use (Table 2).

**Table 2:** Characteristics of the tested STSs

Product name	Origin of the material	Company	Product sold in	Adverse events
Acellular matrices				
AlloDerm®	Human freeze-dried, cell-free, dermal matrix	LifeCell Corp., Branchburg, NJ, USA	USA, distributed in the EU via HTA	None
DynaMatrix®	Porcine small intestinal submucosa (collagens, glycosaminoglycans, glycoproteins, proteoglycans, growth factors)	Keystone Dental, Turnpike Burlington, MA, USA	USA and Europe since 2008	None
Mucograft®	Porcine bilayer collagen matrix	Geistlich Pharma, Wolhusen, Switzerland	USA and EMEA since 2010	None
Tissue engineered				
CelTx™ (Apligraf®)	Living cellular construct composed of human fibroblasts, keratinocytes, and extracellular matrix proteins on type I bovine collagen	Organogenesis, Canton, MA, USA	FDA approved, but not distributed for dental use	24 patients reported >1 adverse event (total of 43 events, no event reported by >2 patients); 3 patients reported adverse events at LCC site (McGuire et al. 2011) [13]
Dermagraft®	Living human fibroblast-derived dermal substitute	Advanced Tissue Sciences, Inc., La Jolla, CA, USA	Withdrawn from the market	None

*FDA* Food and Drug Administration, *HTA* Human Tissue Authority, *EMEA* Europe, Middle East, Africa

#### Type of reported outcome variables

Apart from KT width, only two studies (I, VI) assessed graft contraction quantitatively (Table 3). Regarding PROMs, most studies performed a qualitative assessment; e.g., only three studies (V, VI, VIII) evaluated specifically tissue color and texture after treatment by scoring clinical photographs or direct clinical examination using calibrated examiners, while in five studies (I–IV, VII), only a description by the authors was given (Table 4). Similarly, the pain associated with the intervention (II, III, VI–VIII) or patient preference regarding the intervention (IV–VIII) was assessed by simply asking the patients (Table 5).

**Table 3:** Values of the width of keratinized tissue (mm) at baseline and final evaluation, postintervention gain (mm), mean difference in gain (mm) between test and control groups, graft contraction (%), and frequency of postintervention KT width ≥2 mm (%)

Study (year)	Group	Baseline (mm)	Final evaluation				Graft contraction (%)	Frequency of postintervention KT width ≥2 mm (range or 95% CI of postintervention KT width)
			Values (mm)	Gain (mm)	Mean difference in gain (mm)	Comparison based on		
Acellular matrices								
Wei et al. (2000) [17]	Test	0.68 ± 0.26^a^	*3.25* ± *0.89* ^a^	*2.59* ± *0.92* ^a^	2.98^c^	*A*, *B*, *C*	*71* ± *10* ^a^	NR
	Control	0.57 ± 0.41^a^	*6.15* ± *0.49* ^a^	*5.57* ± *0.44* ^a^			*16* ± *12* ^a^	NR
Harris (2001) [10]	Test	0.6 ± 0.87^a^	*4.7* ± *1.92* ^a^	4.1 ± 1.79^a^		*A*, C	NR	<100% (range 1.5–8.5 mm)
	Control (FGG)	0.8 ± 0.59^a^	*4.8* ± *1.16* ^a^	4.1 ± 1.25^a^	0.00^c^			100% (range 3.0–6.5 mm)
	Control (CTG)	0.4 ± 0.47^a^	*4.0* ± *0.99* ^a^	3.6 ± 0.82^a^	0.50^c^			100% (range 2.5–5.5 mm)
Nevins et al. (2010) [15]	Test	0.8 ± 0.7^a^	*3.4* ± *0.8* ^a^	*2.6* ± *1.1* ^a^	2.7^c^	*A*, *C*	NR	100% (range 2.5–5.0 mm)
	Control	1.1 ± 1.1^a^	*6.4* ± *0.9* ^a^	*5.3* ± *1.3* ^a^				100% (range 5.0–8.0 mm)
Nevins et al. (2011) [16]	Test	NR	*NR*	2.3 ± 1.1^a^	0.80^c^	*A*, C	NR	NR
	Control	NR	*NR*	3.1 ± 0.6^a^				NR
McGuire & Scheyer (2014) [14]	Test	0.88 ± 0.61^a^	*2.92* ± *0.88* ^a^	*2.04* ^c^	1.61^c^	*B*, *C*	NR	96.67% (95% CI 2.59–3.25 mm)
	Control	0.77 ± 0.68^a^	*4.42* ± *0.64* ^a^	*3.65* ^c^				NR (95% CI 4.18–4.66 mm)
Tissue engineered								
McGuire & Nunn (2005) [11]	Test	1.46 ± 0.91^a^	*2.72* (*2.42–3.03*)^b^	1.26^c^	1.31^c^	*B*	*45.5* (*39.5–51.4*)^b^	NR (95% CI 2.42–3.03 mm)
	Control	1.34 ± 0.97^a^	*3.91* (*3.61–4.22*)^b^	2.57^c^			*21.8* (*15.9–27.7*)^b^	NR (95% CI 3.61–4.22 mm)
McGuire et al. (2008) [12]	Test	1.07 (0.89–1.25)^b^	*2.40* (*2.08–2.72*)^b^	*1.33* (*0.95–1.71*)^b^	1.96^c^	*A*, *B*, *C*	NR	76% (95% CI 2.08–2.72 mm)
	Control	1.17 (0.99–1.35)^b^	*4.46* (*4.14–4.78*)^b^	*3.29* (*2.91–3.68*)^b^				100% (95% CI 4.14–4.78 mm)
McGuire et al. (2011) [13]	Test	1.41 ± 0.72^a^	*3.21* ± *1.14* ^a^	1.80^c^	1.34^c^	*B*	NR	95.3% (NR)
	Control	1.43 ± 0.69^a^	*4.57* ± *1.00* ^a^	3.14^c^				NR

Italic values indicate significant difference (*p* < 0.05)

*CTG* connective tissue graft, *FGG* free gingival graft, *KT* keratinized tissue, *NR* not reported, *SD* standard deviation, *A* comparisons between baseline and final evaluation values, *B* comparisons between groups regarded values of KT width at the final evaluation, *C* comparisons between groups regarded values of KT width gain

^a^Mean (±SD)

^b^Mean (95% CI)

^c^Mean (calculation based on the presented data)

**Table 4:** Tissue color and texture in STS and FGG groups at final evaluation

Study (year)	Group	Tissue color			Tissue texture		
		Less	Equally	More	Less	Equally	More
		Red (%)			Firm (%)		
McGuire & Nunn (2005)^a^ [11]	STS	*9.1*	*90.9*	*0.0*	*9.1*	*90.9*	*0.0*
	FGG	*68.2*	*27.3*	*4.6*	*77.3*	*22.7*	*0.0*
McGuire et al. (2011)^b^ [13]	STS	*2.4*	*92.9*	*4.7*	*0.0*	*95.3*	*4.7*
	FGG	*72.9*	*27.1*	*0.0*	*45.9*	*54.1*	*0.0*
		Match to neighboring tissue (%)					
McGuire & Scheyer (2014) [14]	STS	*87*			*97*		
	FGG	*10*			*0*		
		Authors’ description of the STS group^c^					
Wei et al. (2000) [17]		“Appears similar to the alveolar mucosa”			“Appears similar to the alveolar mucosa”		
Harris et al. (2001) [10]		NR			“CTG and AD seemed to produce a more aesthetic result in most cases; however, both produced a result that was as ‘patch like’ in appearance as a FGG”		
McGuire et al. (2008) [12]		“Significant better matching”			“Significant better matching”		
Nevins et al. (2010) [15]		“Excellent color blend”			“Excellent texture blend”		
Nevins et al. (2011) [16]		“Excellent color blend”			“Excellent texture blend”		

Italic values indicate significant difference between the test and control groups (*p* < 0.05)

*AD* Alloderm®, *CTG* connective tissue graft, *FGG* free gingival graft, *NR* not reported

^a^Recorded 12 months after treatment

^b^Recorded 6 months after treatment

^c^Data and evaluation parameters are not presented

**Table 5:** Patient-reported outcome measures on pain level and preference/satisfaction

Study (year)	Group	Pain level				Patient preference/satisfaction
		None (%)	Mild (%)	Moderate (%)	Severe (%)	
McGuire & Nunn (2005)^a^ [11]	STS	13.6	50.0	31.8	4.6	9.91 ± 1.54^b^
	FGG	13.6	54.6	27.3	4.6	10.20 ± 1.13^b^
		After 3 days (%)		After 7 days (%)		
McGuire et al. (2011)^c^ [13]	STS	70.6		45.9		*76.5%*
	FGG	62.3		37.7		*23.5%*
		Authors’ description^d^				
Harris (2001) [10]	STS	“Higher pain levels in the FGG group from the donor site... These patients tended to take more pain medication and for a longer period of time.”				NR
	FGG					
	CTG					
McGuire et al. (2008) [12]	STS	“Subject perception of the duration of pain was reduced in the STS sites.”				*60%*
	FGG					*20%* (no preference *20%*)
Nevins et al. (2010) [15]	STS	“Patients reported less discomfort related to the palatal harvest with the DynaMatrix when compared to the autogenous sites.”				NR
	FGG					
McGuire & Scheyer (2014) [14]	STS	NR				*70%*
	FGG					*30%*
Nevins et al. (2011) [16]	STS	NR				Authors’ description^d^
	FGG					“Significant bias toward avoiding palatal harvesting, in favor of the STS group”
Wei et al. (2000) [17]	STS	NR				NR
	FGG					

Italic values indicate significant difference between STS and FGG groups (*p* < 0.05)

*CTG* connective tissue graft, *FGG* free gingival graft, *NR* not reported, *SD* standard deviation

^a^Pain level at 3 months after treatment (=first evaluation time point)

^b^Mean (±SD) of a specific not clearly defined scale

^c^Pain at recipient site

^d^Data and evaluation parameters are not presented

### Results on KT width

Five studies [RoB: low (VII); unclear (I); high (II–IV)] presented a significant increase in KT width from baseline to final evaluation values (i.e., comparison “A” in Table 3). In the STS and FGG groups, the increase in KT width ranged from 1.26 to 4.1 mm and from 2.57 to 5.57 mm, respectively. Five studies [RoB: low (V, VII, VIII); unclear (I); high (VI)] found significantly wider KT in the FGG group at final evaluation (i.e., comparison “B” in Table 3). Six studies compared gain in KT width between the groups (i.e., comparison “C” in Table 3); four studies [RoB: low (V, VII), unclear (I); high (III)] showed significantly larger gain in the FGG group. Regarding presence of ≥2 mm KT after treatment, three studies [RoB: low (VII); high (II, III)] reported a frequency of 100% for FGG, four studies [RoB: low (V, VII, VIII); high (III)] reported a frequency of 76–100% for STSs, and one study [high RoB (II)] reported a range of 1.5–8.5 mm KT width, i.e., <100% frequency, for STS. Graft contraction was reported in two studies [RoB: unclear (I); high (VI)] (Table 3) and was significantly higher (2 and 4.4 times, respectively) in the STS group.

### Results on PROMs

In all studies, but one (II), significantly better color and texture match of the grafted region with the neighboring tissues was reported for the STS group compared to the FGG group, both when judged by the patients [RoB: low (V, VIII)); high (VI)] or the authors [RoB: low (VII); unclear (I); high (III, IV)]. In general, color and texture match was achieved in about 90% of the cases in the STS group, while the tissue color was less red and the texture was less firm in the grafted area in >70 and >45% of the cases, respectively, in the FGG group (Table 4). In the last study [high RoB (II)], the authors judged the appearance of the STS also as patch like, similarly to the FGG group.

No significant difference between STS and FGG regarding the level of pain experienced by the patient was reported in two studies [RoB: low (VIII); high (VI)]; however, one (VI) of the studies assessed pain only after 3 months postoperatively. In all studies, but one [high RoB (VI)], a significant difference in favor of the STS (range 60–76.5%) regarding patient preference was reported [low RoB (V, VII, VIII)] (Table 5).

### Synthesis of results

#### KT width

##### Pooled comparisons (Fig. 1a)

Regarding the comparison between any kind of STS and FGG, the overall pooled estimate (i.e., not considering the time point of comparison) was −1.55 mm favoring FGG (*p* < 0.001), but with significant heterogeneity. In the sensitivity analysis where *r* was set at an extreme *r* = 0, overall pooled estimate remained significant in favor of FGG with −1.55 mm (95% CIs −1.90, −1.20, *p* < 0.001). The predictive intervals for the overall pooled estimate indicated that the KT width achieved with STS in a future trial is likely to be −2.57 to −0.54 mm less than what would be achieved with FGG.

**Fig. 1:**
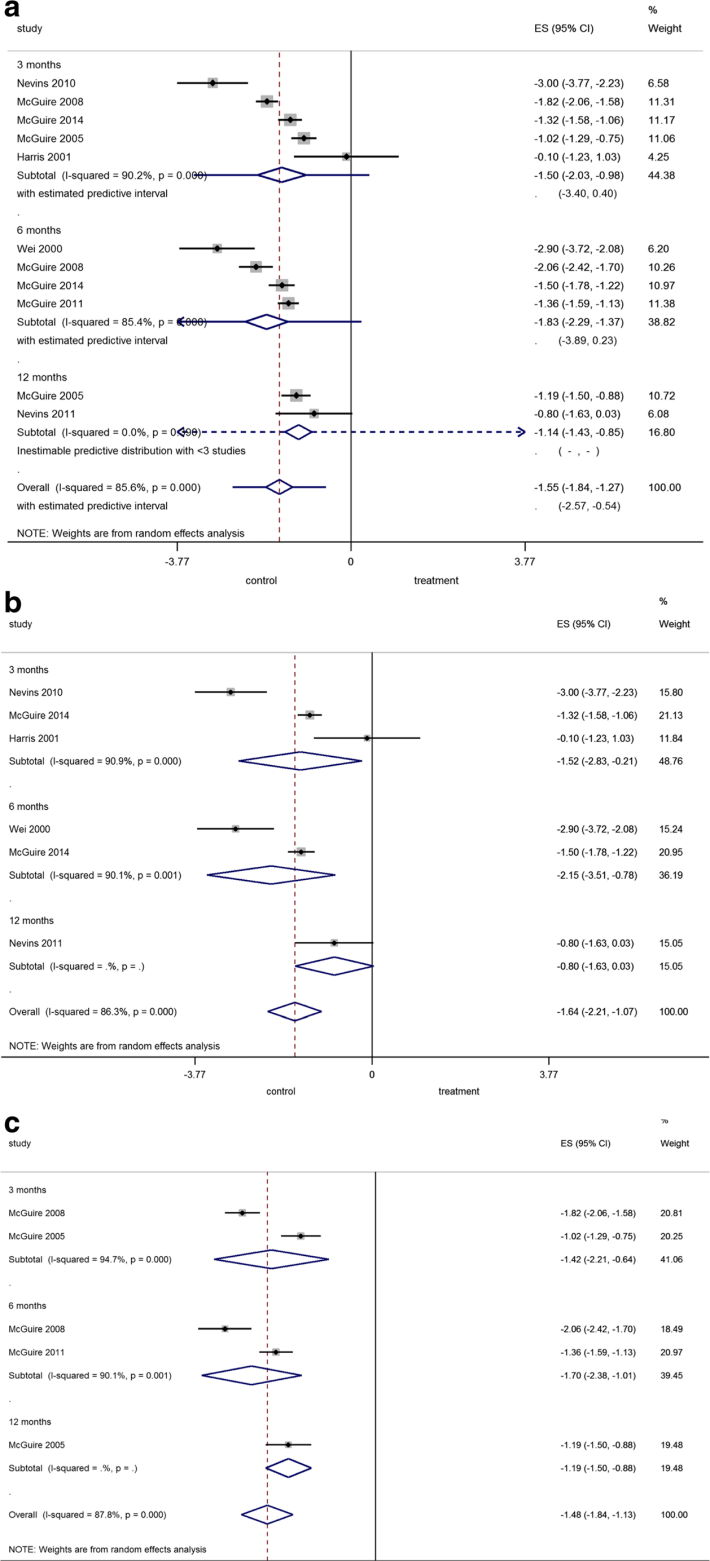
**a–c** Forest plot on the effect size of treatment after application of a FGG (=control) compared to **a** all tested graft substitutes, **b** an acellular matrix, or **c** a tissue-engineered STS (=treatment) overall and after 3, 6, and 12 months

##### Comparisons between FGG and acellular matrices (Fig. 1b)

For comparison between FGG and acellular matrices, the overall pooled estimate was −1.64 mm favoring FGG (*p* < 0.001), but with significant heterogeneity. In the sensitivity analysis where *r* was set at an extreme *r* = 0, overall pooled estimate remained significant with −1.61 mm (95% CIs −2.33, −0.90, *p* < 0.001).

##### Comparisons between FGG and tissue-engineered STSs (Fig. 1c)

For comparison between FGG and tissue-engineered STSs, the overall pooled estimate was −1.48 mm favoring FGG (*p* < 0.001), but heterogeneity was significant. In the sensitivity analysis where *r* was set at an extreme *r* = 0, overall pooled estimate remained significant with −1.48 mm (95% CIs −1.83, −1.12, *p* < 0.001).

#### Graft contraction

Since the last review [3], no new data were available on the parameter “graft contraction.” There, it was reported that STSs showed significantly larger (28.4% on average) contraction compared to that observed in FGG.

#### Tissue color and texture match (Fig. 2a, b)

The overall pooled estimate of the OR for tissue color match in the grafted region with the neighboring tissue was 37.84 favoring STSs (*p* < 0.001); heterogeneity was low. Similar, the overall pooled estimate of the OR for tissue texture match was 70.12 again favoring STSs (*p* < 0.001); heterogeneity was significant.

**Fig. 2:**
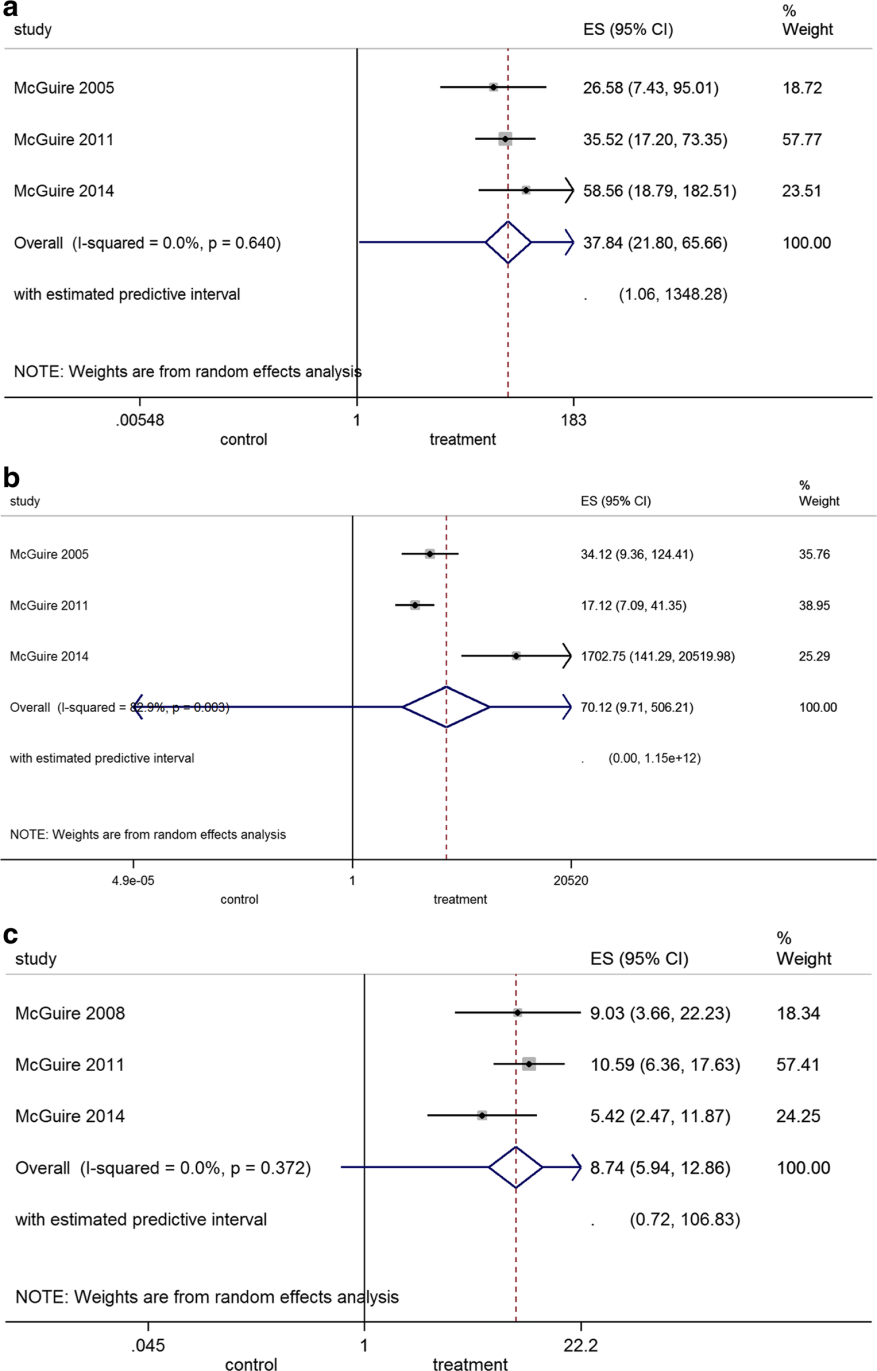
**a–c** Forest plot on the tissue **a** color and **b** texture match and **c** patient preference after application of a FGG (=control) compared to a STS (=treatment)

#### Patient preference (Fig. 2c)

The overall pooled estimate of the OR was 8.74 favoring STSs (*p* < 0.001); heterogeneity was low.

### RoB assessment

Since less than ten studies were included in the meta-analysis, standard funnel plots and contour-enhanced funnel plots [18] were not possible to use to examine publication bias. Three studies (V, VII, VIII) were assessed as of low, one study (I) of unclear, and four studies (II, III, IV, VI) of high RoB. RoB analysis of each of the included studies and overall risk are presented in Appendixes 4 and 5. Further, four studies (I, II, III, IV) were described by the authors as RCTs, but the randomization process was not defined. Reasons for assigning “other bias” to the various studies are also included in Appendix 4.

## Discussion

The results of the present systematic review indicate that, on the basis of relatively limited clinical evidence, the use of a STS is inferior than the use of a FGG harvested from the palate in increasing the width of KT in non-root coverage procedures, when combined with APF. However, better color and texture match of the grafted area with the neighboring tissues is consistently observed with STSs.

The necessity or not to have a minimum amount of AG in order to sustain periodontal health has been debated in the past [19–26]. In particular, based on the observation made in a clinical study that despite daily (professionally delivered) prophylaxis, plaque-free tooth surfaces with <2 mm of KT continued to exhibit clinical signs of inflammation, it was widely suggested that ≥2 mm KT is a requirement for periodontal tissue stability [21]. In other clinical studies, however, patients with limited amount of KT (even with <1 mm) did not experience any attachment loss over a longer period of time [19, 22]. In context, in a systematic evaluation employing a preclinical in vivo model [25, 26], it was demonstrated that periodontal tissues can be maintained clinically and histologically inflammation free, irrespective of the presence or absence of a wide zone of KT, provided that effective plaque control is performed; in contrast, in the presence of plaque, inflammation is clinically (but not histologically) more pronounced at sites with a narrow zone of KT, compared to sites with wide and firm AG. Nevertheless, it has also been reported that, in patients failing to attend supportive periodontal treatment on a regular basis, sites with a narrow zone of KT width (i.e., 1.4 mm on average) presented with an increased gingival index and lost attachment over a period of 6 years, although of questionable clinical magnitude (i.e., 0.5 mm); in contrast, contralateral sites previously augmented and presenting a wide zone of KT did not show any deterioration of their periodontal conditions [27]. Altogether, surgical augmentation of the width of KT in non-root coverage procedures has nowadays rather limited indications; as already mentioned, it was suggested in a recent consensus conference that only in patients where plaque control is inadequate and/or submarginal restoration margins are necessary, soft tissue augmentation procedures should be considered for sites lacking 2 mm KT width (for review, see Scheyer et al. [2]).

Although the use of FGG in combination with APF has been proven to be a predictable technique for increasing KT width on the long term [28, 29], the drawbacks associated with the procedure (i.e., second surgical site; limited supply; surgical complications; often unsatisfactory aesthetic outcome) have generated the pursuit of STSs. Indeed, various types of STSs have been proposed and evaluated in the clinic; these include allogeneic and xenogeneic collagen-based matrices (AlloDerm®, DynaMatrix®, Mucograft®) and tissue-engineered constructs including allogeneic cells seeded in xenogeneic matrices (CelTx™, Dermagraft®). The rationale of using tissue-engineered STSs is that the transplanted cells, which are not supposed to survive at the recipient site, provide a superior wound healing environment by secreting various anti-inflammatory cytokines and growth factors, including pro-angiogenic factors [30–33]. The results of the present meta-analysis revealed that use of a STS results in about 1.1–2.2 mm less KT width increase compared to the use of a FGG. Further, use of a tissue-engineered STS was apparently not superior to the use of an acellular matrix. Specifically, average KT gain after the use of tissue-engineered STSs was never >2mm [11–13], while it ranged between 2.0 and 4.1 mm after the use of acellular matrices [10, 14–17]. This underperformance of STS compared to FGG is also depicted by the significantly larger (by 28%) contraction of STS compared to that of FGG [3]. The results herein indicated also that the use of an STS does not predictably result in a KT width ≥2 mm after treatment. Only in one [15] out of four reporting studies, 100% of the sites treated with STS showed ≥2 mm of KT, while in all three reporting studies, 100% of the sites treated with FGG had ≥2 mm KT width [10, 15]. In perspective, despite the fact that the rationale for performing an augmentation procedure is to achieve KT width ≥2 mm, only half of all included studies reported on the frequency of this outcome.

On the other hand, all but one [10] of the included studies revealed that better tissue color and texture match of the grafted site with the neighboring tissue was achieved with the use of a STS compared to that of a FGG [11–17]. Specifically, color and texture match was achieved in about 90% of the cases treated with a STS, while color and texture mismatch occurred in >70 and >45%, respectively, of the cases treated with a FGG. This finding of poor tissue color and texture match after the use of FGG is by far not surprising, since it is for long known that FGG preserves the histological characteristics of the donor site after transplantation [34]. Similarly, most studies reporting on patient preference described a significant difference in favor of the use of STS [12–14], while only one study [11] described no difference. Indeed, no remarkable adverse reactions were observed, thus raising no safety concerns for the use of STS. It seems reasonable to assume that patients favored STS due to less discomfort and/or pain compared to the use of a FGG. Nevertheless, in the two studies [11, 13], where pain was assessed using a validated instrument, no difference was recorded between the treatment groups. It has, however, to be mentioned that the use of a split-mouth design (as several studies herein [11–14]) may bring bias in pain assessment [35–37]. Again, it is interesting to note that despite the fact that less discomfort and/or pain and better aesthetic results are among the incentives to use STS instead of a FGG, these parameters were systematically and/or properly evaluated only in a fraction of the included studies.

In addition, limited standardization and large variability were observed among the studies regarding various factors related to the surgical procedure, e.g., the size of the recipient bed and/or application of single or multiple STS layers, which appear to influence the outcome and might be responsible for the significant heterogeneity that was frequently observed. Particularly, improved results in KT width gain have been reported with increased mesio-distal graft dimension [12] (i.e., treatment of multiple teeth) and the use of a multi-layer technique [11]. Thus, comparisons among studies, regarding the performance of the different STSs, have to be done with caution. Furthermore, when judging the currently available evidence on the topic, one has to take into account that only three of the included studies [12–14] where judged as of low RoB. It is thus reasonable to require that future studies consistently and systematically follow the CONSORT guidelines for reporting of RCTs [38] and evaluate and report on the possible effect of anatomical and surgical factors (e.g., size of the recipient bed and vestibulum depth; treatment of single or multiple sites; application of single or multiple layers) and on relevant treatment outcomes (i.e., frequency of ≥2 mm KT width postoperatively; PROMs).

In summary, the present systematic review and meta-analyses reached basically to similar conclusions as previous systematic reviews [3, 39] on with this topic:

- No preclinical in vivo studies comparing autogenous soft tissue grafts with a STS material are available.
- Use of STSs (acellular matrix or tissue engineered) in combination with APF resulted in a significantly less gain of KT width compared to what achieved with FGG and APF.
- Use of a tissue-engineered STS was apparently not superior to the use of an acellular matrix.
- Use of STS does not predictably result in a KT width ≥2 mm after treatment, while use of FGG does.
- Significantly better aesthetic outcomes and larger patient preference in favor of STS were observed.
- STS materials appeared to be safe.

## Electronic supplementary material

Appendix 1 Prisma 2009 checklist. (DOC 66 kb)

Appendix 2 Flowchart of the inclusion process of studies for the systematic review. (GIF 59 kb)

High Resolution Image (TIFF 6648 kb)

Appendix 3 Reasons for exclusion of 33 full-texts (6 preclinical in vivo and 27 human trials). (DOCX 17 kb)

Appendix 4 Risk of bias of included studies according to the Cochrane Collaboration′s Tool. (DOCX 68 kb)

Appendix 5 Overall risk of the included studies on non-root coverage procedures to increase the width of keratinized tissue. (GIF 30 kb)

High Resolution Image (TIFF 2881 kb)
