# Supplementary figures and images for: Soft tissue substitutes in non-root coverage procedures: a systematic review and meta-analysis
Source: Clin Oral Investig. 2017 Jan 20;21(2):505–18. doi: 10.1007/s00784-016-2044-4 (PMC5318480; doi:10.1007/s00784-016-2044-4)

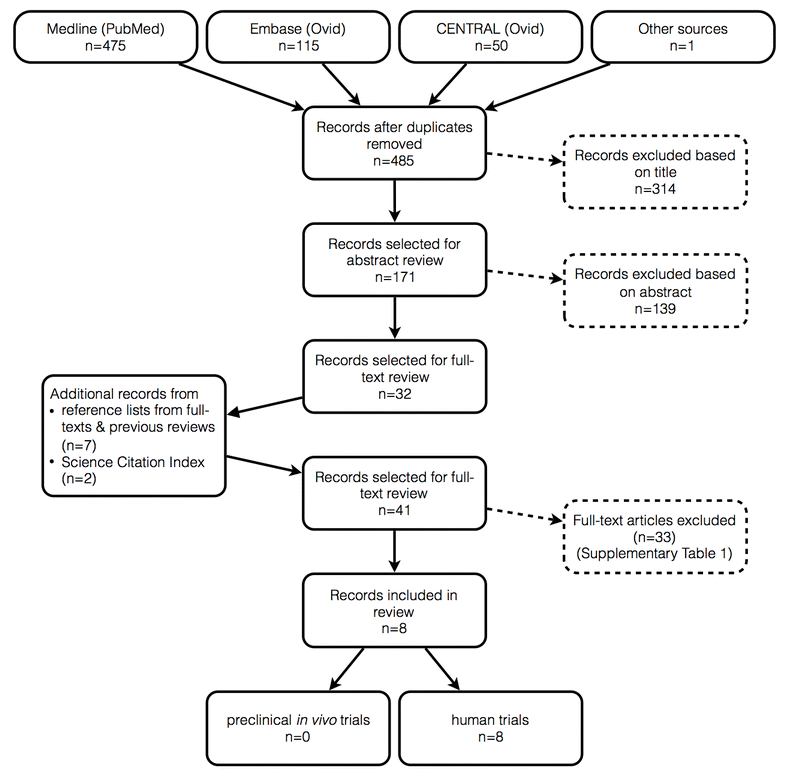

Supplement: Supplementary file 2 — Flowchart of the inclusion process of studies for the systematic review. (GIF 59 kb) [file 784_2016_2044_Fig3_ESM.gif]

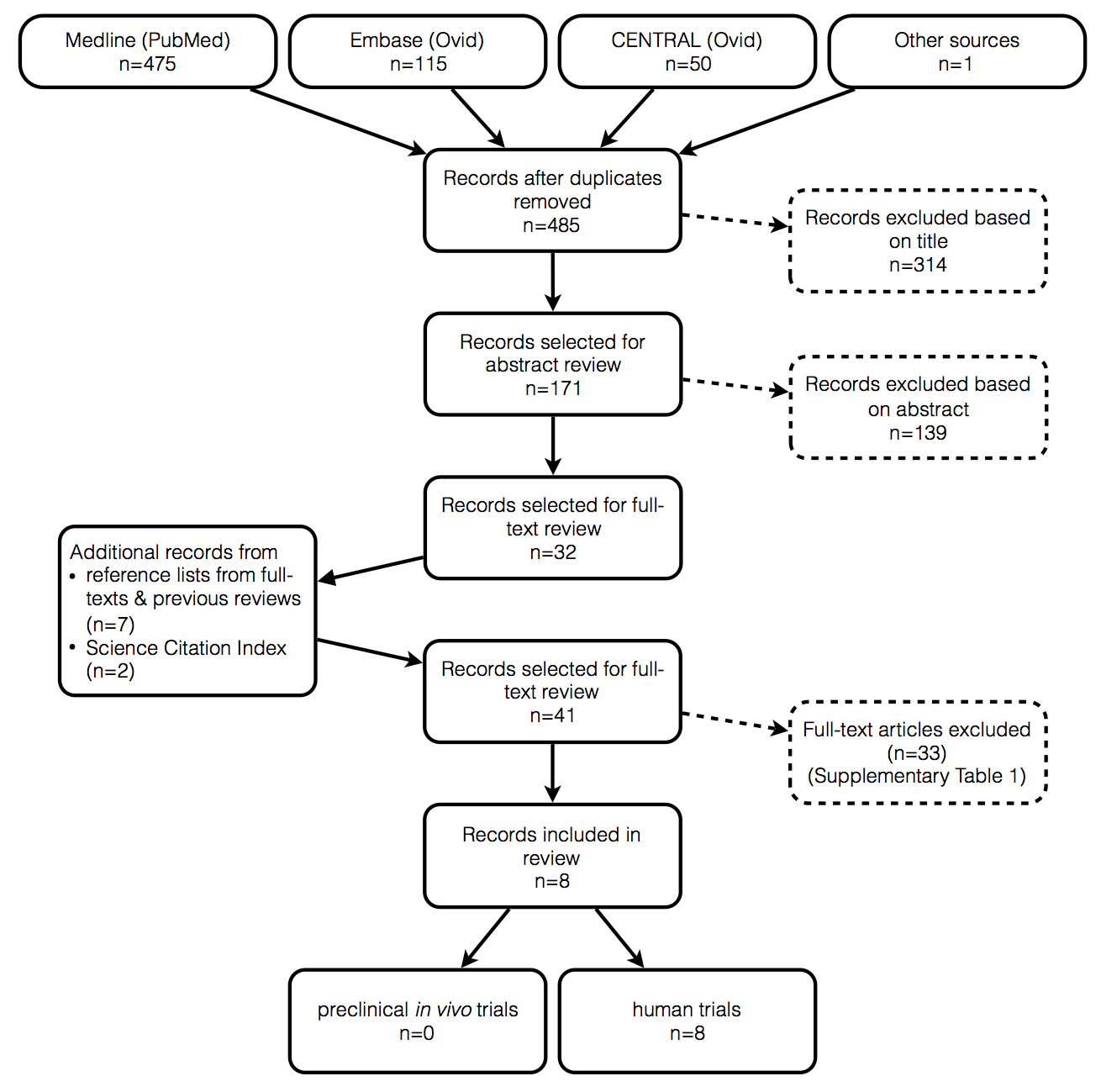

Supplement: Supplementary file 3 — High Resolution Image (TIFF 6648 kb) [file 784_2016_2044_MOESM2_ESM.tiff]

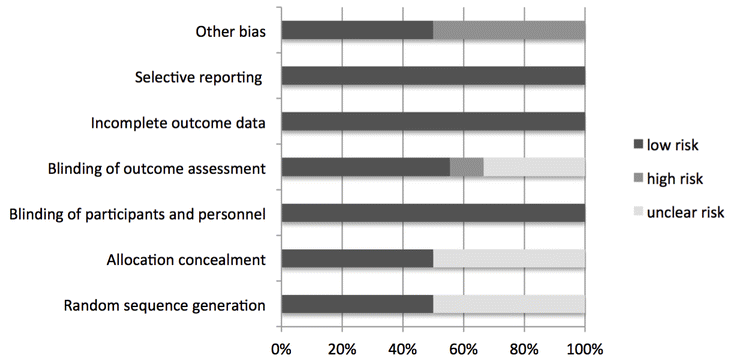

Supplement: Supplementary file 6 — Overall risk of the included studies on non-root coverage procedures to increase the width of keratinized tissue. (GIF 30 kb) [file 784_2016_2044_Fig4_ESM.gif]

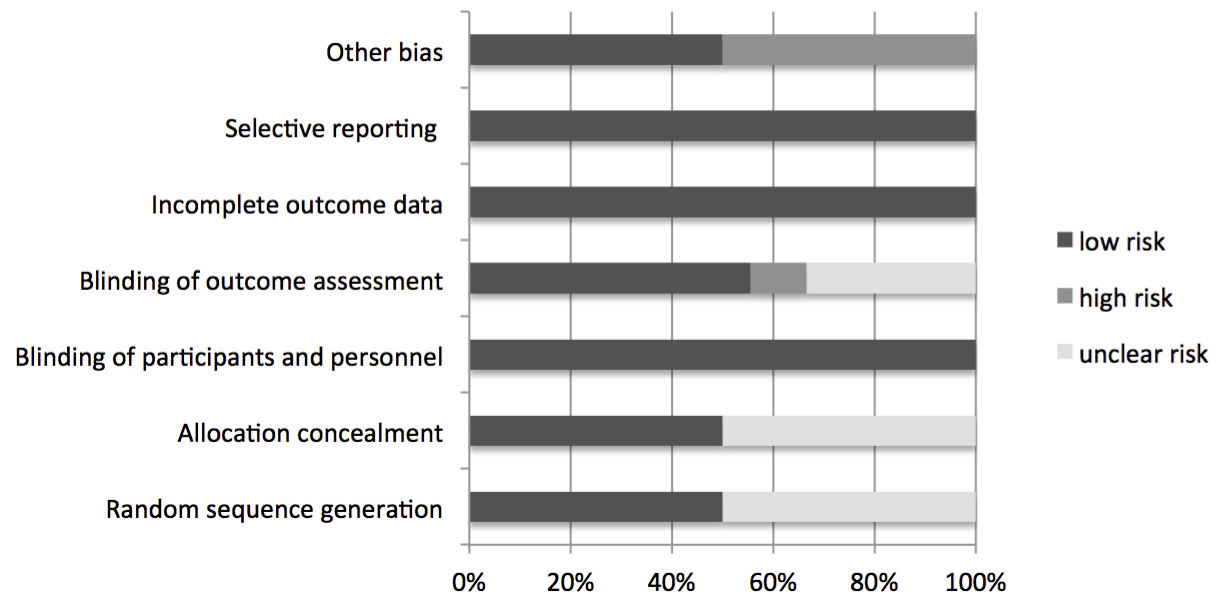

Supplement: Supplementary file 7 — High Resolution Image (TIFF 2881 kb) [file 784_2016_2044_MOESM5_ESM.tiff]
