# Supplementary material for: Soft tissue substitutes in non-root coverage procedures: a systematic review and meta-analysis
Source: Clin Oral Investig. 2017 Jan 20;21(2):505–18. doi: 10.1007/s00784-016-2044-4 (PMC5318480; doi:10.1007/s00784-016-2044-4)
Supplement: Supplementary file 4 — Reasons for exclusion of 33 full-texts (6 preclinical in vivo and 27 human trials). (DOCX 17 kb) [file 784_2016_2044_MOESM3_ESM.docx]

**Appendix S3.** Reasons for exclusion of 33 full-texts (6 preclinical *in vivo* and 27 human trials).

| **Study (year)** | **Reason for exclusion** |
| --- | --- |
| *Preclinical in vivo trials* |  |
| Carroll et al. 1974 [1] | No control group with autologous tissue |
| Yukna & Sullivan 1978 [2] | Only histological assessment |
| Novaes et al. 2007 [3] | No control group with autologous tissue |
| Jung et al. 2011 [4] | No control group with autologous tissue |
| Lotfi et al. 2011 [5] | No control group with autologous tissue |
| Vignoletti et al. 2014 [6] | No control group with autologous tissue |
| *Human trials* |  |
| von Weyhrother et al. 1972 [7] | No control group with autologous tissue |
| Köster & Flores de Jacoby 1973 [8] | No data presented |
| Krekeler 1974 [9] | No control group with autologous tissue |
| Bernimoulin et al. 1975 [10] | No FGG substitute tested |
| Schoo & Coppes 1976 [11] | No preoperatively values recorded |
| Yukna et al. 1977 [12] | Less than 5 patients |
| Yukna et al. 1977 [13] | No control group with autologous tissue |
| Matter 1979 [14] | No FGG substitute tested |
| Bartolucci 1981 [15] | No control group with autologous tissue |
| Ouhayoun et al. 1983 [16] | No controlled study design, only 2 patients treated with FGG |
| Shulman 1996 [17] | No control group with autologous tissue |
| Callan & Silverstein 1998 [18] | No control group with autologous tissue, single case report |
| Haeri et al. 1999 [19] | No control group with autologous tissue |
| Haeri & Parsell 2000 [20] | Single case report |
| Wei et al. 2002 [21] | The same patients (Wei et al. 2000) |
| Sezer et al. 2004 [22] | Only edentulous patients |
| Mohammadi et al. 2007 [23] | No control group with autologous tissue |
| Sanz et al. 2009 [24] | Results for implants and teeth are not presented separately |
| Scarano et al. 2009 [25] | No control group with autologous tissue |
| Vieira et al. 2009 [26] | No control group with autologous tissue |
| Nevins 2010 [27] | No control group with autologous tissue, less than 5 patients |
| Morelli et al. 2011 [28] | No clinical parameters measured, only 4 weeks follow-up |
| Dominiak et al. 2012 [29] | No control group with autologous tissue |
| George et al. 2012 [30] | No FGG substitute tested, single case report |
| Izumi et al. 2013 [31] | No control group with autologous tissue |
| Scheyer et al. 2014 [32] | The same patients (McGuire et al. 2008, McGuire et al. 2011) |
| Yadav et al. 2014 [33] | No FGG substitute tested |

FGG, free gingival graft.
