# Supplementary material for: Soft tissue substitutes in non-root coverage procedures: a systematic review and meta-analysis
Source: Clin Oral Investig. 2017 Jan 20;21(2):505–18. doi: 10.1007/s00784-016-2044-4 (PMC5318480; doi:10.1007/s00784-016-2044-4)
Supplement: Supplementary file 5 — Risk of bias of included studies according to the Cochrane Collaboration′s Tool. (DOCX 68 kb) [file 784_2016_2044_MOESM4_ESM.docx]

**Supplementary Table 3.** Risk of bias of included studies according to the Cochrane Collaboration`s Tool.

| **Study (year)** | **Random sequence generation** | **Allocation concealment** | **Blinding of participants and personnel** | **Blinding of outcome assessment** | **Incomplete outcome data** | **Selective outcome reporting** | **Other bias (reasons for assigning "other bias")** | **Overall risk of bias** |
| --- | --- | --- | --- | --- | --- | --- | --- | --- |
| Wei et al. (2000) | ? | ? | + | + | + | + | + | **?** |
| Harris (2001) | ? | ? | + | - | + | + | -  (no information on smoking status) | **-** |
| McGuire & Nunn (2005) | + | + | + | + | + | + | -  (test sites treated with different amount of layers of the graft substitute) | **-** |
| McGuire et al. (2008) | + | + | + | + | + | + | + | **+** |
| Nevins et al. (2010) | ? | ? | + | ? | + | + | -  (no information on smoking status) | **-** |
| Nevins et al. (2011) | ? | ? | + | ? | + | + | -  (no information on smoking status) | **-** |
| McGuire et al. (2011) | + | + | + | + | + | + | + | **+** |
| McGuire & Scheyer (2014) | + | + | + | + | + | + | + | **+** |

„+“, low risk; „-“, high risk; „?“; unclear risk.
